# Efficacy of Combined Intravitreal Bevacizumab and Dexamethasone Compared to Bevacizumab Alone for Treatment of Persistent Macular Edema Secondary to Retinal Vein Occlusion

**DOI:** 10.18502/jovr.v21.18760

**Published:** 2026-10-26

**Authors:** Saeed Karimi, Fatemeh Farahani, Seyed-Hashem Daryabari, Seyyed-Morteza Hosseini Imeni

**Affiliations:** ^1^Ophthalmic Research Center, Research Institute for Ophthalmology and Vision Science, Shahid Beheshti University of Medical Sciences, Tehran, Iran; ^2^Department of Ophthalmology, Labbafinejad Medical Center, Shahid Beheshti University of Medical Sciences, Tehran, Iran; ^3^Dida Eye Research Center, Bushehr University of Medical Sciences, Bushehr, Iran

**Keywords:** Bevacizumab, Dexamethasone, Macular Edema, Retinal Vein Occlusion, Visual Acuity

## Abstract

**Purpose:**

This study evaluated the short-term anatomical and visual outcomes and complications of simultaneous intravitreal injection of bevacizumab and dexamethasone versus bevacizumab alone in patients with persistent macular edema caused by retinal vein occlusion (RVO) admitted at Labbafinejad Medical Center.

**Methods:**

This prospective interventional study was performed on 50 eyes from 50 patients with RVO-related macular edema refractory to at least three monthly bevacizumab injections. The eligibility criteria included central macular thickness (CMT) > 300 µm and < 10% reduction from baseline. Patients were alternately allocated to two groups: one received monthly bevacizumab (1.25 mg) alone (monotherapy group) and the other received intravitreal bevacizumab combined with dexamethasone (200 µg) for three sessions (combination group). Best-corrected visual acuity (BCVA), CMT, and intraocular pressure (IOP) were measured at baseline and 1 month after the third injection.

**Results:**

Baseline CMT was 511.4 ± 139.05 µm in the combination group, which decreased to 417.58 ± 156.72 µm 1 month after the last injection (*P* = 0.004), while the reduction of CMT was not significant in the monotherapy group. In comparison, combination therapy yielded a significantly greater CMT reduction than bevacizumab monotherapy (*P* = 0.020). BCVA improved significantly from baseline only in the combination group (*P* = 0.016), though the difference in visual improvement between groups was not significant (*P* = 0.457). In patients with central retinal vein occlusion (CRVO), CMT reduction was notably greater with combination therapy (*P* = 0.015) compared to branch retinal vein occlusion (BRVO). Visual and anatomical improvements were comparable between BRVO and CRVO subgroups. Combination therapy did not increase the risk of elevated IOP.

**Conclusion:**

For treatment-resistant macular edema secondary to RVO, simultaneous intravitreal bevacizumab and dexamethasone could reduce CMT and improve BCVA more effectively than bevacizumab alone, without increasing IOP. Given the cost and limited access to dexamethasone implants, adjunctive intravitreal dexamethasone solution is a viable option for persistent macular edema secondary to RVO.

##  INTRODUCTION

Retinal vein occlusion (RVO) is the second most prevalent vascular cause of retinal disorders.^[1]^ Macular edema (ME) is recognized as a significant complication of RVO.^[2]^ About 5-15% of patients with branch retinal vein occlusion (BRVO) develop ME within 1 year, while most patients with central retinal vein occlusion (CRVO) present with ME at the onset of the disease.^[3]^ The pathophysiology of ME due to RVO is complex; however, studies show that vascular endothelial growth factor (VEGF) and other growth factors, such as placental growth factor and platelet-derived growth factor, as well as pro-inflammatory cytokines including interleukin-6 and interleukin-8, play a significant role in its severity.^[4]^

Current treatment of ME due to RVO involves intravitreal injection of anti-VEGF agents, which are commonly employed as the initial treatment option.^[5]^ Intravitreal steroids target the inflammatory component of ME pathophysiology.^[2]^ Corticosteroids suppress the expression of prostaglandins, cytokines, and growth factors, including VEGF.^[6]^ They also inhibit the activity, adhesion, and migration of leukocytes.^[7]^ Moreover, by acting on the basement membrane of the retinal capillary endothelium and strengthening tight intercellular junctions, corticosteroids reduce vascular permeability and prevent fluid leakage into the retinal interstitium.^[8]^ Therefore, intravitreal corticosteroids are used alone or in combination with anti-VEGF therapy and are usually reserved for unresponsive cases because of the potential for cataract formation and increased intraocular pressure (IOP).^[2]^

Dexamethasone is seven times more potent than triamcinolone acetate and is less lipophilic compared to triamcinolone, which results in reduced deposition within the trabecular meshwork and crystalline lens. Consequently, it carries a lower risk of increased IOP and cataract progression.^[9]^

While intravitreal dexamethasone was initially thought to have a limited duration of action, anatomical and visual improvements have been observed to persist for at least 1 month.^[10]^ The intravitreal dexamethasone implant (700 µg), marketed as Ozurdex, is a sustained-release corticosteroid system designed to reduce the frequency of intraocular steroid injections.^[9]^ It has been shown that the Ozurdex implant leads to long-term improvement in vision and ME over a 3-year period.^[11]^ Although effective, the widespread use of intravitreal dexamethasone implants remains limited by high cost and restricted availability.^[10]^ Dexamethasone solution presents a more economical and widely accessible option, costing about US$2, compared to dexamethasone implants, which are priced at approximately US$1475. Multiple studies have demonstrated its efficacy in reducing central macular thickness (CMT) and enhancing visual acuity in individuals with diabetic ME.^[10,11,12]^

Due to the limited data on dexamethasone treatment of persistent ME in RVO cases, we conducted this study to assess the short-term effectiveness and safety of a single intravitreal dexamethasone solution injection as a cost-effective and readily available treatment alternative to dexamethasone implant, combined with bevacizumab, in reducing CMT and improving visual acuity in patients with persistent ME secondary to RVO.

##  METHODS

This study was designed as a prospective interventional study. Patients with RVO and persistent ME who presented to the retina clinic at Labbafinejad Hospital were included. The inclusion criteria were age over 18 years, best-corrected visual acuity (BCVA) < 20/40, a history of at least three consecutive monthly intravitreal bevacizumab injections with a minimum of 6-week interval since the last injection, and a CMT > 300 µm with < 10% reduction from baseline. The study was conducted in accordance with the Declaration of Helsinki, and the study protocol was approved by the Ethics Committee of Shahid Beheshti University of Medical Sciences (Ethical code: IR.SBMU.MSP.REC.1403.472). All patients were enrolled after providing comprehensive informed consent, and no additional costs were incurred by the participants. The exclusion criteria included a history of intravitreal steroid injection within the past 3 months, intraocular surgery within the past 3 months, prior vitrectomy, history of glaucoma or use of IOP-lowering medications, IOP > 21 mmHg, vitreous hemorrhage, concurrent diabetic retinopathy, BCVA < 20/400, positive relative afferent pupillary defect (RAPD), and media opacities such as cataract.

Patients were allocated to two groups in an alternating sequence. The first group (combination) received three monthly injections of intravitreal dexamethasone 200 µg/0.05 ml (Dexon 8 mg/2 ml ampoule, Sinadarou, Iran) in addition to intravitreal bevacizumab 1.25 mg/0.05 ml (Stivant 100 mg/4 ml vial, CinnaGen, Iran). The dexamethasone dose was selected to avoid the need for anterior chamber paracentesis. The second group (monotherapy) received the standard treatment of bevacizumab 1.25 mg/0.05 ml administered monthly for 3 months. Patients were examined prior to the initiation of the treatment protocols and again 1 month after the final intravitreal injection. Each examination included BCVA measurement, a comprehensive slit-lamp examination, IOP measurement using the Goldmann tonometer, fundus examination, and optical coherence tomography (OCT; Heidelberg Engineering, Heidelberg, Germany).

The primary outcomes of this study were defined as changes in CMT and BCVA measured 1 month after the last injection compared to baseline.

All examinations were conducted by the same attending vitreoretinal physician, and imaging was performed by the same technician in every case. Additionally, all images were evaluated by the same attending vitreoretinal specialist.

### Outcome Measures

Changes in BCVA and CMT were the primary outcome measures of this study. The secondary outcome measures included rise of IOP and any other adverse events.

Sample Size

The minimum sample size was calculated based on a two-tailed independent-samples *t*-test, with reference to the study by Maturi et al.^[13]^ Considering an effect size of 1.326, a type I error rate ( α ) of 0.05, a power (1– β ) of 0.95, and an allocation ratio of 1:1, the estimated sample size was 16 patients per group. To account for potential loss to follow-up, we increased the final sample size to 31 patients per group and accordingly enrolled 31 patients in the combination group and 31 in the monotherapy group.

### Statistical Analysis

Mean, standard deviation, mode, range, and frequency (percentage) were used to describe the data. For categorical variables, the chi-square test and Fisher's exact test were applied. For numerical data with a normal distribution, the *t*-test was used, while the Mann–Whitney U test was applied for nonparametric data.

All analyses were conducted using SPSS version 25 (IBM Corp., Armonk, New York, USA), with a *P*-value < 0.05 considered statistically significant.

##  RESULTS

A total of 62 eyes from 62 patients were enrolled in this study. During the study, 12 patients were lost to follow-up. Finally, 20 patients in the combination group and 30 patients in the monotherapy group completed the study.

The mean age was 63.2 ± 8.42 years in the combination group and 61.66 ± 8.7 years in the monotherapy group. Males comprised 70% of both groups (Combination: 14, Monotherapy: 21). In the combination group, 12 patients (60%) had a history of BRVO, compared to 22 patients (73.3%) in the monotherapy group. The remaining patients in both groups had a history of CRVO. Regarding lens status, 14 patients (70%) in the combination group and 26 patients (86.6%) in the monotherapy group were phakic, with the rest being pseudophakic.

Seven patients (35%) in the combination group and 15 (50%) in the monotherapy group had a history of diabetes. A history of hypertension was more common in the monotherapy group (63.3%) compared to the combination group (40%).

The two groups did not differ significantly regarding any of the demographic parameters [Table 1].

At baseline, the BCVA measured 0.75 ± 0.36 LogMAR (logarithm of the minimum angle of resolution) in the combination group and 0.77 ± 0.33 LogMAR in the monotherapy group. One month following the final injection, BCVA improved to 0.68 ± 0.40 LogMAR in the combination group and was 0.74 ± 0.32 LogMAR in the monotherapy group [Table 2]. The monotherapy group showed no significant change in BCVA (*P* = 0.460), while the combination group experienced a statistically significant improvement (*P* = 0.016). However, the differences in terms of baseline and final BCVA between the groups did not reach statistical significance (*P *= 0.857 and *P* = 0.597, respectively). Furthermore, the difference in BCVA improvement between the groups was not significant either (*P* = 0.457).

Baseline CMT was 511.4 ± 139.05 µm in the combination group and 497.04 ± 128.59 µm in the monotherapy group. As indicated in Table 3, no statistically significant difference was found between the two groups (*P* = 0.727).

One month after the last injection, CMT decreased to 417.58 ± 156.72 µm in the combination group and 488.13 ± 165.81 µm in the monotherapy group. While this reduction was not statistically significant in the monotherapy group (*P* = 0.718), it was statistically significant in the combination group (*P* = 0.004), as detailed in Figure 1. Moreover, the reduction in CMT was significantly greater in the combination group compared to the monotherapy group (*P* = 0.020).

We observed a more significant reduction in CMT among patients with hypertension receiving combination therapy in the combination group compared to monotherapy in the monotherapy group (*P* = 0.004). In the combination therapy group, no meaningful differences were detected between male and female patients in the extent of CMT reduction and BCVA improvement [Table 4]. However, in the monotherapy group, women experienced significantly greater improvement in BCVA than men (*P* = 0.02); this difference may be attributed to the smaller number of women compared to men in the group. More accurate interpretation and conclusions will require future studies with larger sample sizes. Furthermore, patients with CRVO showed a more significant reduction in CMT with combination therapy [Figure 2] compared to monotherapy (*P* = 0.01), as detailed in Table 5. The reduction in CMT did not differ significantly between BRVO and CRVO patients treated with combination therapy (*P* = 0.620). Other factors such as phakic or pseudophakic status had no significant effect on the primary outcomes of the study [Table 6].

During the study, we observed no major complications related to the intravitreal injection, including endophthalmitis, uveitis, retinal detachment, cataract formation, or vitreous hemorrhage. Additionally, no cases of increased IOP were reported [Table 7].

**Table 1:** Baseline characteristics of patients in the two treatment groups

		**IVB + Dexa (** ***N*** ** = 20)**	**IVB (** ***N*** ** = 30)**	***P*** **-value**
Age (yr)	Mean ± SD	63.2 ± 8.42	61.66 ± 8.7	0.540 ${}^{**}$
Gender	Male	14 (70%)	21 (70%)	0.448 ${}^{*}$
	Female	6 (30%)	9 (30%)	
Laterality	OD	6 (30%)	12 (40%)	0.470 ${}^{*}$
	OS	14 (70%)	18 (60%)	
Retinal vein occlusion	BRVO	12 (60%)	22 (73.3%)	0.322 ${}^{*}$
	CRVO	8 (40%)	8 (26.7%)	
Lens type	Phakic	14 (70%)	26 (86.7%)	0.171 ${}^{**}$
	Pseudophakic	6 (30%)	4 (13.3%)	
Underlying disease	DM	7 (35%)	15 (50%)	0.295 ${}^{*}$
	HTN	8 (40%)	19 (63.3%)	0.105 ${}^{*}$
	IHD	1 (5%)	5 (17.2%)	0.379 ${}^{***}$
${}^{*}$ Chi-square test; ${}^{**}$ Independent *t*-test; ${}^{***}$ Fisher's exact test. IVB, intravitreal bevacizumab; yr, year; Dexa, dexamethasone; SD, standard deviation; DM, diabetes mellitus; HTN, hypertension; IHD, ischemic heart disease.				

**Table 2:** Baseline and final BCVA values

	**IVB**	**IVB+DEXA**	***P*** **-value**
Baseline BCVA (LogMAR)	0.77 ± 0.33	0.75 ± 0.36	0.857
Final BCVA (LogMAR)	0.74 ± 0.32	0.68 ± 0.40	0.597
BCVA change	–0.03 ± 0.20	–0.06 ± 0.11	0.457
*P-*value	0.460	0.016	
IVB, intravitreal bevacizumab; Dexa, dexamethasone; BCVA, best corrected visual acuity; LogMAR, logarithm minimum angle of resolution.			

**Table 3:** Baseline and final CMT values

	**IVB**	**IVB+ Dexa**	***P-*** value**
Baseline CMT ( μ m)	497.04 ± 128.59	511.4 ± 139.05	0.727
Final CMT ( μ m)	488.13 ± 165.81	417.58 ± 156.72	0.167
CMT change	–8.91 ± 116.94	–93.82 ± 135.08	0.020
*P*-within ${}^{*}$	0.718	0.004	
IVB, intravitreal bevacizumab; Dexa, dexamethasone; CMT, central macular thickness.			

**Table 4:** Changes in BCVA and CMT by gender in the two treatment groups

**Group**	**Change in**	**Male**	**Female**	***P*** -value*
IVB + Dexa	CMT	–120.93 ± 149.66	–52.80 ± 71.00	0.308
	BCVA	–0.08 ± 0.12	0.00 ± 0.00	0.072
IVB	CMT	–3.71 ± 112.25	–23.67 ± 139.62	0.726
	BCVA	0.02 ± 0.18	–0.14 ± 0.17	0.020
${}^{*}$ Mann–Whitney nonparametric test; IVB, intravitreal bevacizumab; Dexa, dexamethasone; BCVA, best corrected visual acuity; CMT, central macular thickness.				

**Figure 1:**
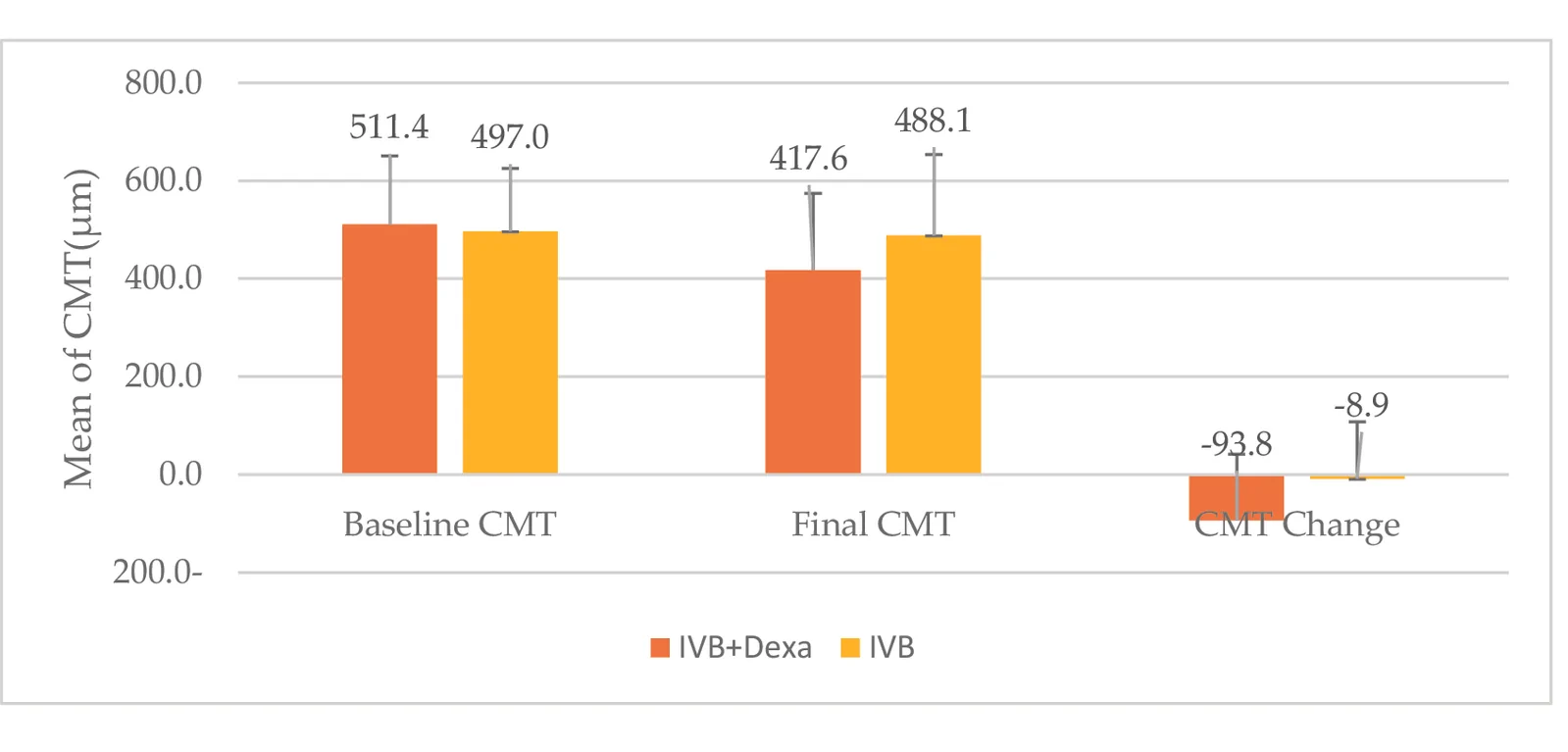
Comparison of baseline and final CMT and the change in CMT between the two treatment groups.

**Figure 2:**
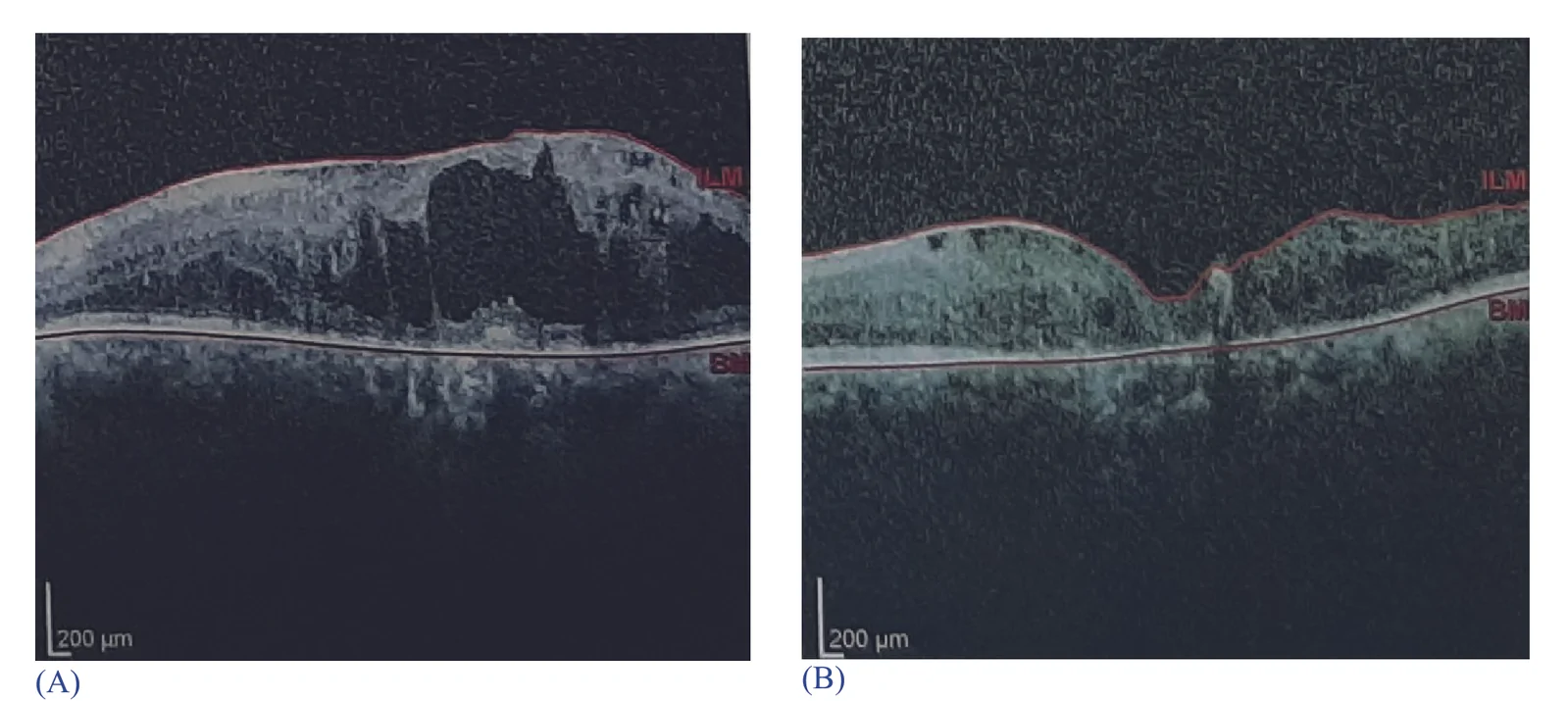
Optical coherence tomography (OCT) images from a 70-year-old male patient with CRVO in the combination therapy group, who had previously received 16 intravitreal bevacizumab injections without noticeable improvement: (A) marked central macular edema and outer retinal disruption at baseline (OS); (B) a substantial decrease in central macular thickness 1 month after the final intravitreal injections of bevacizumab and dexamethasone.

**Table 5:** Comparison of changes in CMT, BCVA, and IOP in patients with BRVO and CRVO between the two treatment groups

**Type**	**Change in**	**IVB + Dexa**	**IVB**	***P*** **-value**
BRVO	CMT	–73.55 ± 112.54	–39.56 ± 95.61	0.393
	BCVA	–0.05 ± 0.11	–0.04 ± 0.21	0.843
CRVO	CMT	–143.5 ± 159.96	101.4 ± 130.08	0.015
	BCVA	–0.08 ± 0.10	0.01 ± 0.14	0.169
BRVO, branch retinal vein occlusion; CRVO, central retinal vein occlusion; IVB, Intravitreal bevacizumab; Dexa, Dexamethasone; BCVA, best corrected visual acuity; CMT, central macular thickness.				

**Table 6:** Comparison of BCVA, CMT, and IOP in phakic and pseudophakic patients within the two treatment groups

**Group**	**Change in**	**Pseudophakic**	**Phakic**	***P*** -value*
IVB + Dexa	CMT	–151.8 ± 151.51	–85.57 ± 130.23	0.405
	IOP	0.33 ± 0.52	–0.71 ± 2.02	0.104
	BCVA	–0.02 ± 0.04	–0.08 ± 0.12	0.224
IVB	CMT	–54.00 ± 45.25	–4.62 ± 121.31	0.383
	IOP	0.00 ± 1.41	–0.50 ± 2.77	0.590
	BCVA	–0.13 ± 0.15	–0.01 ± 0.20	0.167
*Mann–Whitney nonparametric test; IVB, intravitreal bevacizumab; Dexa, dexamethasone; BCVA, best corrected visual acuity; CMT, central macular thickness; IOP, intraocular pressure.				

**Table 7:** Comparison of baseline, final, and change in intraocular pressure between the two treatment groups

**IOP (mmHg)**	**IVB**	**IVB + Dexa**	***P*** -value*
Baseline	13.97 ± 1.97	14.5 ± 2.37	0.392
Final	13.53 ± 2.29	14.1 ± 2.57	0.418
Change	–0.43 ± 2.62	–0.4 ± 1.76	0.960 ${}^{**}$
*P*-within ${}^{*}$	0.373	0.322	
${}^{*}$ Paired *t*-test; ${}^{**}$ Independent *t*-test for difference in changes; IVB, intravitreal bevacizumab; Dexa, dexamethasone; IOP, intraocular pressure.			

##  DISCUSSION

In this prospective interventional study, we found that adding intravitreal dexamethasone to anti-VEGF therapy (bevacizumab) significantly reduced CMT in patients with RVO and persistent ME, compared with intravitreal bevacizumab alone.

Spooner et al reported that although switching to aflibercept may reduce CMT in resistant cases, visual improvement may not correlate with CMT reduction in these patients.^[14]^ Karimi et al demonstrated that combining intravitreal bevacizumab and dexamethasone solution significantly enhanced both visual and anatomical outcomes without increasing the risk of elevated IOP in diabetic patients with anti-VEGF-resistant ME, compared to treatment with bevacizumab alone.^[10]^ Our study also showed that in patients with CRVO, combination therapy resulted in a significantly greater reduction in CMT compared to monotherapy. Additionally, the reduction in CMT showed no significant variation between BRVO and CRVO patients within the combination group. Nevertheless, as this finding is exploratory, studies with larger sample sizes are needed to draw more definitive conclusions. Neri et al assessed the effectiveness of combining dexamethasone phosphate solution with aflibercept compared to aflibercept alone in patients with DME. Their findings revealed that 1 month following the third injection, the average decrease in CMT was significantly larger in the group receiving combination therapy (176 ± 129 μ m) versus those treated with aflibercept monotherapy (54 ± 49 μ m; *P *= 0.034). Furthermore, BCVA showed greater improvement in the combination group, while the monotherapy group exhibited only a slight change. IOP remained stable across both groups, without any statistically significant difference (*P *= 0.855).^[12]^ Jirarattanasopa et al evaluated the efficacy of intravitreal dexamethasone implants in patients with retinal vascular disorders accompanied by ME. Their study demonstrated significant improvement in BCVA and CMT 6 months after treatment. Additionally, the study revealed that patients with ME resulting from RVO experienced greater improvement in BCVA compared to patients with DME.^[15]^ The COMRADE-C study compared a single injection of sustained-release dexamethasone implant to monthly and PRN ranibizumab and observed greater BCVA gains and CMT reductions with ranibizumab. However, it is important to interpret these results with caution because the single dexamethasone implant injection was administered during the study period.^[16]^ Limon conducted a non-randomized prospective study to compare the effectiveness of a slow-release intravitreal dexamethasone implant combined with bevacizumab against bevacizumab alone in persistent DME. The combination therapy group experienced significant gains in BCVA, improving from a mean of 44 ± 19 ETDRS letters at baseline to 59 ± 18 letters at 1 month (*P* < 0.05), 57 ± 19 letters at 2 months (*P* < 0.05), and 55 ± 18 letters at 3 months (*P* < 0.05). In contrast, the monotherapy control group did not demonstrate any significant changes in BCVA. Furthermore, the combination group showed a mean reduction of 142.25 μ m in CMT over 3 months (*P* < 0.05), whereas the monotherapy group had a nonsignificant decrease of 17.30 μ m during the same period (*P* > 0.05).^[17]^ In our study, there was no significant difference in BCVA change between the two groups. However, while the monotherapy group did not experience significant improvement in BCVA, the combination group showed a significant improvement, which may correlate with the reduction in CMT observed in these patients.

Current evidence indicates that approximately half of patients with BRVO and less than half of those with CRVO achieve complete resolution of ME within 5 years of intravitreal anti-VEGF therapy.^[18]^ Several proinflammatory cytokines and growth factors, other than VEGF, have been identified as elevated in patients with RVO and resultant ME.^[4]^ These factors should be considered especially in persistent cases, since anti-VEGF injections do not address all contributors to ME, whereas steroid injections have been shown to reduce both proinflammatory cytokines and VEGF levels.^[16]^ Campochiaro et al analyzed 21 pro-permeability proteins before and after intravitreal dexamethasone implant injection in patients with RVO and found variable responses of these proteins to the treatment. They also observed that no single protein could be identified as a definitive co-contributor alongside VEGF in the pathogenesis of ME.^[19]^ Zhang et al demonstrated that combining intravitreal anti-VEGF therapy with steroids enhances both visual and anatomical outcomes and prolongs the injection-free interval in patients with ME secondary to RVO.^[2]^ A meta-analysis by Qiongzhen et al found that dexamethasone implants benefit patients with refractory ME by improving BCVA and CMT, with effects lasting approximately 6 months.^[20]^ These findings underscore the complex pathophysiology of ME in patients with RVO and, as suggested in the aforementioned studies, dexamethasone injections can be considered a multi-targeted approach in managing ME. Our observations align with prior studies showing significantly greater CMT reduction in patients receiving the combination therapy compared to the monotherapy group. This highlights the importance of considering factors beyond VEGF in the pathogenesis of refractory ME.

Aside from the risks of retinal tears, detachments, and endophthalmitis generally associated with intravitreal injections, intravitreal corticosteroids have been shown to carry additional risks, including steroid-induced cataract formation in phakic patients and increased IOP, which may lead to glaucoma.^[16]^ Haller et al noted that repeated dexamethasone implant injections were associated with predictable adverse events, and IOP spikes were typically manageable with IOP-lowering medications.^[21]^ No serious adverse events were observed among the study participants, and no instances of elevated IOP following intravitreal injections were detected throughout the study period. Notably, our combination group included a relatively high number of phakic patients who did not develop visually significant cataracts following injections. Similarly, Karimi et al and Neri et al reported no significant change in IOP in either group.^[10,12]^ However, the short follow-up duration represents a limitation of these studies, particularly regarding the effects of intravitreal dexamethasone injection on the risk of cataract progression and elevated IOP. To ensure a more accurate assessment of the safety profile of this treatment approach, studies with larger sample sizes and long-term follow-up are essential.

This study has its limitations, notably the relatively small sample size, high rate of loss to follow-up, and short follow-up duration. Future randomized controlled trials with longer follow-up periods and larger sample sizes are needed to further clarify the optimal steroid treatment regimen for patients with refractory ME.

Although intravitreal dexamethasone injections were once thought to have a limited duration of effect, our findings suggest that combining dexamethasone solution with anti-VEGF therapy provides an effective approach for treating refractory ME, with lower cost and greater accessibility compared to dexamethasone implants. This combined treatment led to significant improvements in BCVA and CMT, at least within 1 month. Further research is needed to determine whether these improvements result from the interaction between the two drugs or primarily from dexamethasone alone. In our study, intravitreal dexamethasone injections were found to be relatively safe, with no patients experiencing IOP spikes or major adverse events. In summary, intravitreal dexamethasone solution could be a safe, effective, and cost-efficient adjunctive therapy for refractory ME secondary to RVO.

### Financial Support and Sponsorship

None.

### Conflicts of Interest

None.
